# Toxic Relationships: Prediction of TBT’s Affinity to the Ecdysteroid Receptor of *Triops longicaudatus*

**DOI:** 10.3390/toxics11110937

**Published:** 2023-11-17

**Authors:** Nuno Gonçalo de Carvalho Ferreira, Adriano Chessa, Isabel Oliveira Abreu, Luís Oliva Teles, Peter Kille, António Paulo Carvalho, Laura Guimarães

**Affiliations:** 1 Interdisciplinary Centre of Marine and Environmental Research (CIIMAR), University of Porto, Terminal de Cruzeiros do Porto de Leixões, Avenida General Norton de Matos, S/N, 4450-208 Matosinhos, Portugalisabellabreu@gmail.com (I.O.A.); loteles@fc.up.pt (L.O.T.); apcarval@fc.up.pt (A.P.C.);; 2 School of Biosciences, Cardiff University, Museum Avenue, Cardiff CF10 3AX, UK; 3 Biology Department, Faculty of Science, University of Porto, Rua do Campo Alegre 687, 4169-007 Porto, Portugal; 4 School of Medicine and Biomedical Sciences, University of Porto, Rua de Jorge Viterbo Ferreira 228, 4050-313 Porto, Portugal

**Keywords:** comparative genomics, tributyltin, proteomics, phylogeny, crustacea

## Abstract

Tributyltin (TBT) is a biocide introduced in the 1960s in antifouling paints. Despite legislation banning its use, its persistence in the environment still causes significant harm to organisms. Tributyltin is a ligand of retinoid X receptors (RXR) and ecdysteroid receptors (EcRs), which in arthropods act as homologs of RXR. Focusing on Metazoan species, this study used genomic and proteomic information from different sources to compare their three-dimensional structure, phylogenetic distribution, and amino acid sequence alterations. The objective was to identify possible patterns that relate organisms’ sensitivity to TBT using the species *Triops longicaudatus* as the basis for the comparisons. The results showed great conservation of this protein across several species when comparing the interaction amino acids described to RXR (an EcR analog) in *Homo sapiens*. The three-dimensional comparison of RXR showed little conformational variation between different sequences by maintaining the interaction pocket. As for the Species Sensitivity Distribution (SSD) curve, an HC_05_ = 0.2649 [0.0789–0.7082] µg/L was obtained with no specific distribution between the different taxa. Protein-ligand docking analysis was then used to confirm the SSD curve ranking of species. Still, the results showed an opposite trend that may be related, for example, to differences in the LC_50_ values used in the calculations. This study serves as the first step for applying bioinformatics techniques to produce information that can be used as an alternative to animal or cellular experimentation. These techniques could be adapted to various chemicals and proteins, allowing for observations in a shorter timeframe and providing information on a broader spectrum.

## 1. Introduction

Tributyltin-based compounds (TBT) appeared in the 1960s when they started to be incorporated in paints and were highly used to eliminate biofouling in vessels’ hulls and underwater pipes and structures [1]. Unfortunately, not much information on TBT persistence and long-term adverse effects was available in those decades, thus leading to its unrestricted use and ubiquitous environmental contamination [2,3]. TBT was finally banned in the European Union in 2008. These compounds may be introduced into the environment by continuous release from treated structures, such as vessels’ hulls and pipes, and wood, paper, and textiles treated with TBT as a preservative [4]. However, despite several efforts and the creation of specific legislation to reduce its usage, measurable concentrations of TBT are consistently found in environmental samples, suggesting illegal or unregulated use, and still detected in leisure boat hulls [5]. For example, environmental concentrations of TBT up to 1540 ng/L were found in the United Kingdom, 262 ng/L in Brazil, and 26.9 ng/L in South Korea [6,7,8]. Besides being persistent in sediments, TBT is also known to bioaccumulate in aquatic organisms, potentially leading to bioamplification throughout the food chain [1]. The negative impact of TBT on the environment is still a hot research topic in field and laboratory studies. TBT’s widespread occurrence in several environmental compartments, its continuous presence due to low degradation rates, and its resuspension from sediments into the water column (for example, due to adverse climatic events that are becoming more frequent) are some of the reasons why studies on TBT continue to be of high importance. Here, it is essential to highlight the low degradation rate for TBT (mainly in sediments) that can last from several years to decades [9]. These sediments that become TBT reservoirs can release, as described before, TBT into the water column due to adverse climate conditions, but they can also be used as fertilisers in agricultural fields, reaching other compartments. This compound is known to cause endocrine disruption, mitochondrial toxicity [10], immune system suppression [11], and developmental/genetic toxicity [3], impacting both invertebrates and vertebrates. TBT targets specific proteins and may form heterodimers with receptors with homologous and orthologous functions in different taxa. In vertebrates, retinoid X receptor (RXR) and the heterodimers it forms with peroxisome proliferator-activated receptors (PPARs) are the primary targets [12]. Instead, insects and crustaceans have homologous proteins with slightly different or additional functions. For example, in insects, the ultraspiracle protein (USP) is an RXR homolog that forms a heterodimer with the ecdysteroid receptor (EcR). RXR proteins are retained in crustaceans and combined with EcR [13,14]. EcR is a nuclear receptor belonging to the well-conserved ecdysteroid regulatory circuit. It is crucial to organisms that undergo moulting processes during their lifespan, with insects and crustaceans being the most emblematic examples. Besides the previously mentioned more evident effects, TBT also severely impacts moulting processes in Arthropods through critical changes in the hormonal pathways [15,16]. The huge amount of of research about TBT has already been performed, though it is mainly related to its effects on vertebrates and molluscs. As a result, the molecular mechanisms of action in some invertebrate groups, like crustaceans, are not yet fully understood.

*Triops longicaudatus* is a freshwater crustacean inhabiting ephemeral or semi-permanent ponds worldwide. It is considered a cryptic species and a living fossil due to its fundamentally unchanged morphological traits for more than 170 million years [17]. Most populations consist of female individuals, so reproduction is mainly asexual, leading to clonal individuals and, ultimately, clonal populations. Equally important is their small size, short generational time, simple maintenance, and easy manipulation, making *T. longicaudatus* a suitable model organism for ecotoxicological experiments [18]. This species also possesses a simple nervous system, unable to feel pain or distress, thereby becoming an attractive alternate model organism in line with the 3Rs (Reduce, Refine, Replace). This species mainly reproduces asexually, resulting in clonal individuals and thus reducing genetic variations during bioassays. *T. longicaudatus* is considered a living fossil as its genetic information and basic morphology have not changed significantly for more than 170 million years, a feature of great value for comparative genomics [18]. The main focus of comparative genomics and similar approaches is to compare different sequences (whole genomes or specific sections) to infer the functional annotation of both genes and genomes and assess variations between those sequences, which can be interspecific or intraspecific [19]. These studies can also be combined with predictions of the three-dimensional structure of proteins, mainly using the information obtained by aligning the sequence of interest of one or more proteins with well-documented structures [20]. This prediction can then be used to quantify the level of affinity between a given molecule or chemical and a specific binding site of a protein, helping to infer its effects at higher organisational levels [21]. Predicting protein sensitivity greatly helps save time and resources by reducing the number of assays required to characterise and assess the toxicity of a chemical.

This study aimed to determine the interaction between *T. longicaudatus* EcR protein and TBT and the possibility of using protein–ligand docking modeling to rank toxicity. To achieve this goal, a Species Sensitivity Distribution (SSD) curve for TBT was built and compared with protein–ligand affinities to determine the more sensitive and resilient species exposed to TBT. 

## 2. Materials and Methods

### 2.1. Organism Culture

An initial clonal culture of *T. longicaudatus* was obtained by hatching commercial cysts (TriopskingTM—Augsburg, Germany). The organisms were kept at 25 ± 1 °C, with a 12 h:12 h (light:dark) photoperiod in an aquarium (40 L) filled with 2 cm of natural sand (SwellReptiles—Cheshire, UK) and dechlorinated water. Nauplii were fed ad libitum with crushed chlorophyll flakes (Tropical^®^). Adults were fed daily with fish granulate (Vitality & Colour from Tropical^®^, Porto Alegre, Brazil). Once a week, the water was changed. Laid cysts were recovered from the sand at least once a month. The collected sand was air-dried to allow desiccation of the resistant cysts for at least 21 days. The cysts were then stored at room temperature in a dry location. For the exposure experiments, the cysts were carefully selected under a stereomicroscope to check for viability and rehydrated for hatching.

### 2.2. Species Sensitivity Distribution

An acute toxicity test was first performed to evaluate the toxicity of TBTO to *T. longicaudatus*, as no data for this species was available in the literature. For this, cysts were hatched, and the organisms were maintained in culture for up to seven days. Five organisms were transferred to each round polyethene test container (36.13 Ø × 4 cm) with 220 mL of the corresponding solution. To avoid loss through the adsorption of TBTO to the test containers, the recipients were bathed with the test solutions for 24 h prior to the exposure. The test solutions were renewed daily. A total of nine concentrations of tributyltin oxide (TBTO) and ten animals per treatment were tested along with the control (0, 0.001, 0.002, 0.004, 0.008, 0.016, 0.032, 0.064, 0.128, and 0.256 mg/L). The final test concentrations were planned from the results of pilot range-finding tests. The exposure was carried out under the same conditions as the cultures: 25 ± 1 °C and a 12 h:12 h (light:dark) photoperiod. Survival was recorded twice a day for the entire exposure duration (96 h). The median lethal concentration (LC_50_) and respective 95% confidence interval (CI95%) were calculated using a four-parameter logistic curve in SigmaPlot^®^. 

Toxicity data from freshwater organisms was obtained from the public database USEPA ECOTOX Knowledgebase (https://cfpub.epa.gov/ecotox/, accessed on 22 May 2023) [22] and used to build a Species Sensitivity Distribution curve (SSD). The search included LC_50_ values of freshwater organisms exposed up to seven days to TBT-based compounds, along with other additional information such as the life stage of the organism, type of exposure, etc. For this study, only LC_50_ values from adults were selected. To calculate the SSD, the SSD Toolbox v.1.0 software was used (https://www.epa.gov/chemical-research/species-sensitivity-distribution-ssd-toolbox, accessed on 22 May 2023) [23]. This software combines several algorithms that aid users in interpreting the SSD and provides different distribution types for different scenarios [24]. A total of 212 LC_50_ values were found for 64 taxa ranging from diatoms to rotifers to vertebrate species. 

### 2.3. Protein Sequence Selection and Phylogenetic Analysis

The NCBI and the UniProt search engines were used to search for high-quality sequences of our target proteins EcR and RXR-α. Proteins consisted of an average of 430 amino acids. Data was imported into Geneious^®^ software v9.1.8 (www.geneious.com) for multiple sequence comparison by log expectation (MUSCLE alignment). A phylogenetic tree was built using the genetic distance model of Jukes–Cantor and the neighbor joining method, with 1000 bootstrap replications. The sequence from Homo sapiens was used as the outgroup.

From the initial 64 taxa used to create the SSD curve, only 16 were used for the subsequent protein modeling. The excluded taxa corresponded to 19 vertebrates and two algae species (only invertebrates were used), one cnidarian and one tunicate (which were only identified up to the genus), and several others with no data available (one cnidarian, ten molluscs, seven arthropods, and three annelids). In addition to the previously mentioned taxa, the *Palaemonetes pugio* and *Elliptio complanata* sequences were excluded due to the short size of their protein sequences, respectively 215 and 180 amino acids. *Tigriopus japonicus* and *Perna viridis* were also excluded as the alignment did not show amino acids in all interaction residues (please see the next section regarding the interaction residues). The selected sequences and accession codes are listed in Table 1.

### 2.4. Protein Modeling and Protein–Ligand Docking

Information regarding the interaction amino acids between the H. sapiens RXR-α and TBT molecule was collected from the PDBsum database (id: 3e94; https://www.ebi.ac.uk/thornton-srv/databases/pdbsum/, accessed on 22 May 2023) and used to determine similarities between the different species. The protein’s interaction amino acids and corresponding positions were VAL265, ILE268, TRP305, ASN306, LEU309, PHE313, ILE324, VAL342, ILE345, PHE346 VAL349, CYS432, HIS435, LEU436, and PHE439. CYS432 is responsible for the covalent bond to the central tin atom of the TBT molecule and is considered the main contributor to the binding strength [25]. 

The sequences were imported into the SWISS-MODEL database (https://swissmodel.expasy.org) to assess the proteins’ three-dimensional conformation in the different taxa. The analysis did not include the insect class since they possess a specific RXR ortholog, the ultraspiracle protein (USP). Models of the proteins were built based on the structure of the most similar template in the database (Table 1). Afterwards, the structure was imported into AutoDockTools (v1.5.7) [26]. The protein–ligand docking with TBT was performed using Autogrid and AutoDock (V4.2.6) [26]. The most appropriate docking was selected based on the lowest free binding energy (in kcal/mol). The 2D receptor–ligand interactions were plotted using Discovery Studio Visualizer (v21.1.0.20298). 

## 3. Results

### 3.1. Species Sensitivity Distribution

**Figure 1:**
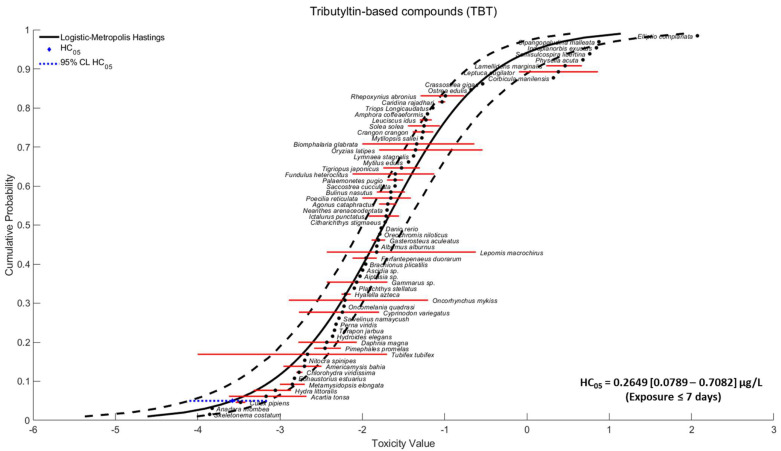
Species Sensitivity Distribution (SSD) curve based on the LC_50_ values of tributyltin-based compounds for aquatic species and corresponding HC_05_. Red lines correspond to the range of LC50 values for that specific species; blue dotted lines correspond to the HC05 range, and black dashed lines correspond to the 95% confidence limit of the model.

The 96 h LC_50_ and CI95% determined for *T. longicaudatus* exposed to TBTO were 0.072 [0.046–0.139] mg/L. An SSD was built using the LC_50_ values of aquatic organisms obtained with exposures of less than seven days (Figure 1). The best fit was determined for the Logistic distribution curve with the Metropolis–Hastings fitting method. The hazardous concentration for 5% of species (HC_05_) predicted from the distribution was 0.2649 [0.0789–0.7082] µg/L (*p* = 0.7862). It was possible to observe that the species were distributed throughout the curve with no evident grouping. The species *Elliptio complanata*, a mollusc, was found to be the most tolerant to TBT-based compounds, while *Skeletonema costatum*, a diatom, was the most sensitive species. The three species below the HC_05_ were, in increasing order of sensitivity: *Culex pipiens* (arthropod), *Anadara rhombea* (mollusc), and *Skeletonema costatum* (diatom).

### 3.2. Sequence Alignments and Phylogenetic Analysis

**Figure 2:**
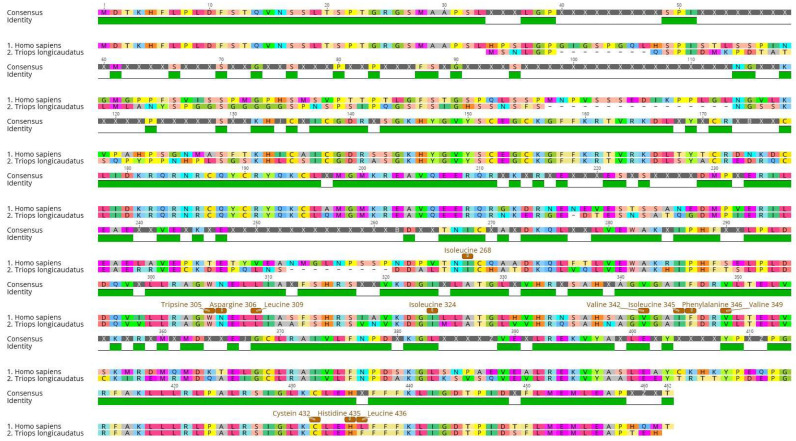
Sequence alignment of retinoid X receptor-α isoform from *Homo sapiens* and *Triops longicaudatus* showing consensus amino acids. Interaction residues and their position are identified in brown in the *Homo sapiens* sequence.

The alignment (Figure 2) provides insight into the conservation level of targeted amino acids between *H. sapiens* and *T. longicaudatus* sequences. The conservation around these specific amino acids was relatively high, with the difference, being that LEU265 replaces VAL265 and PHE436 replaces LEU436. 

**Figure 3:**
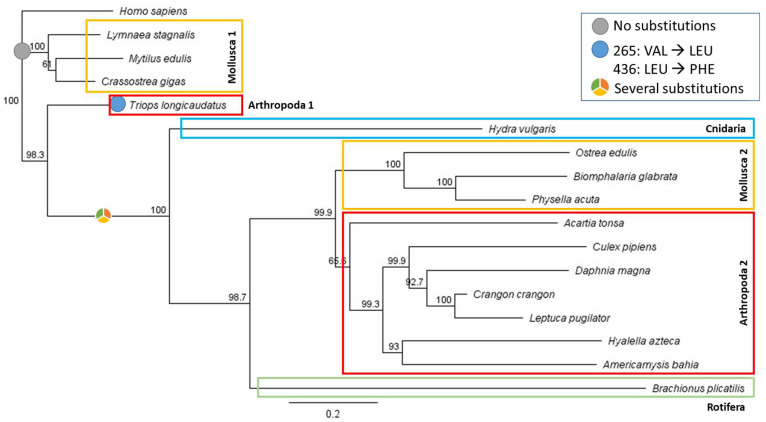
Neighbour joining phylogenetic tree from the different species’ proteins. Bootstrap values are shown at the beginning of each branch. The genetic distance can be assessed through branch lengths. Different coloured circles represent changes in the interaction amino acids compared to the *Homo sapiens* sequence.

In amino acid replacement cases, low values in Grantham’s distance matrix, such as LEU/VAL (score of 32) and LEU/PHE (score of 22), reflect a low evolutionary distance. When analysing the conservation of interaction amino acids between the human RXR-α and the other species (Table 2), huge differences were observed even within species of the same taxa. The alignment showed that three species of molluscs (*Lymnaea stagnalis*, *Mytilus edulis,* and *Crassostrea gigas*) had identical interaction amino acids in all positions that contrasted with the remaining molluscs where only ILE324 and LEU436 were identical to the human RXR-α. *T. longicaudatus* also showed almost the same conservation of interaction amino acids for arthropods, whereas most other arthropods showed different amino acids. Finally, the numbers of identical residues to human RXR-α for the cnidarian and rotifer species were restricted to three and one, respectively. A phylogenetic tree to evaluate evolutionary distance is presented in Figure 3. As expected, the phylogenetic tree presented a species clustering according to their taxa, except for molluscs (two branches are shown) and the branch that separated *T. longicaudatus* from the other arthropods.

### 3.3. Protein Modeling

**Figure 4:**
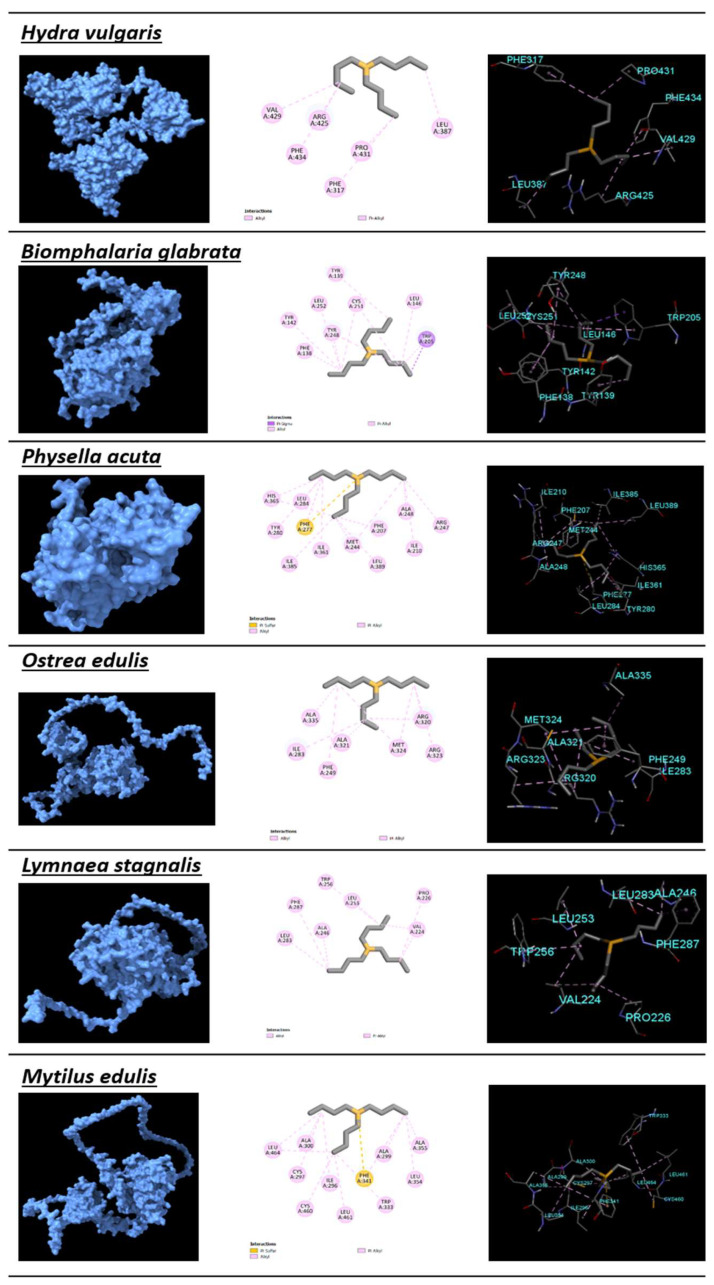

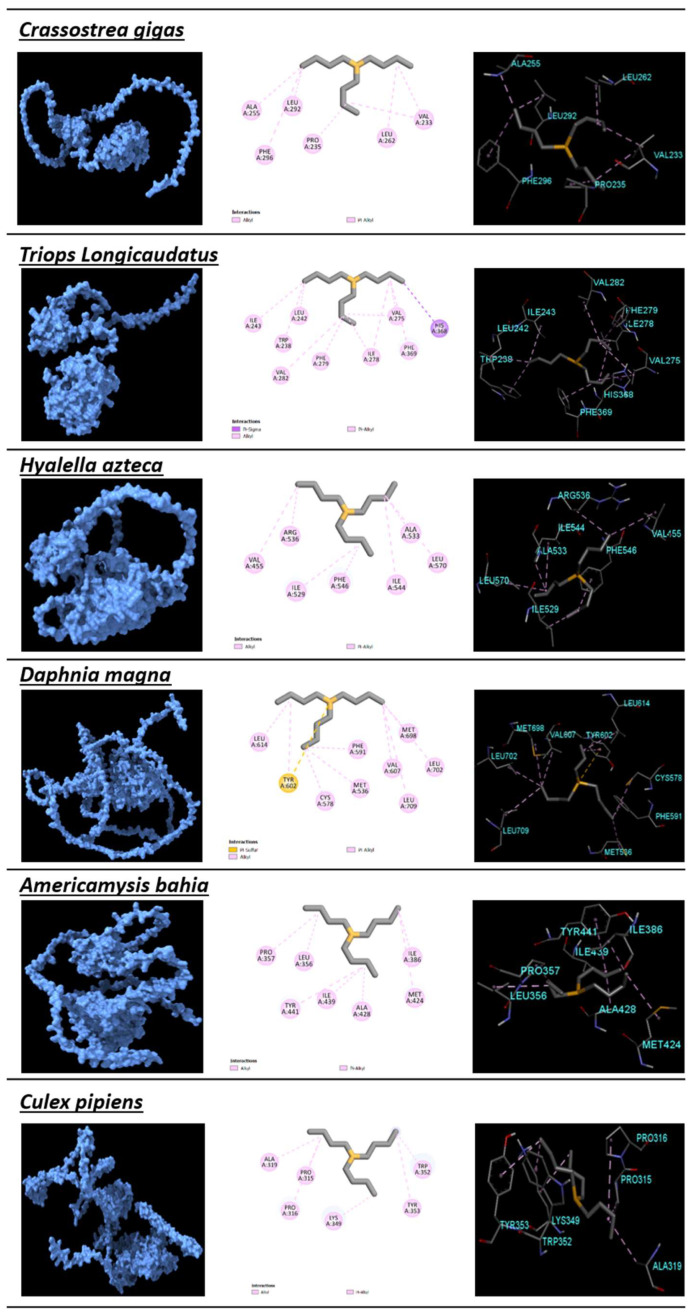

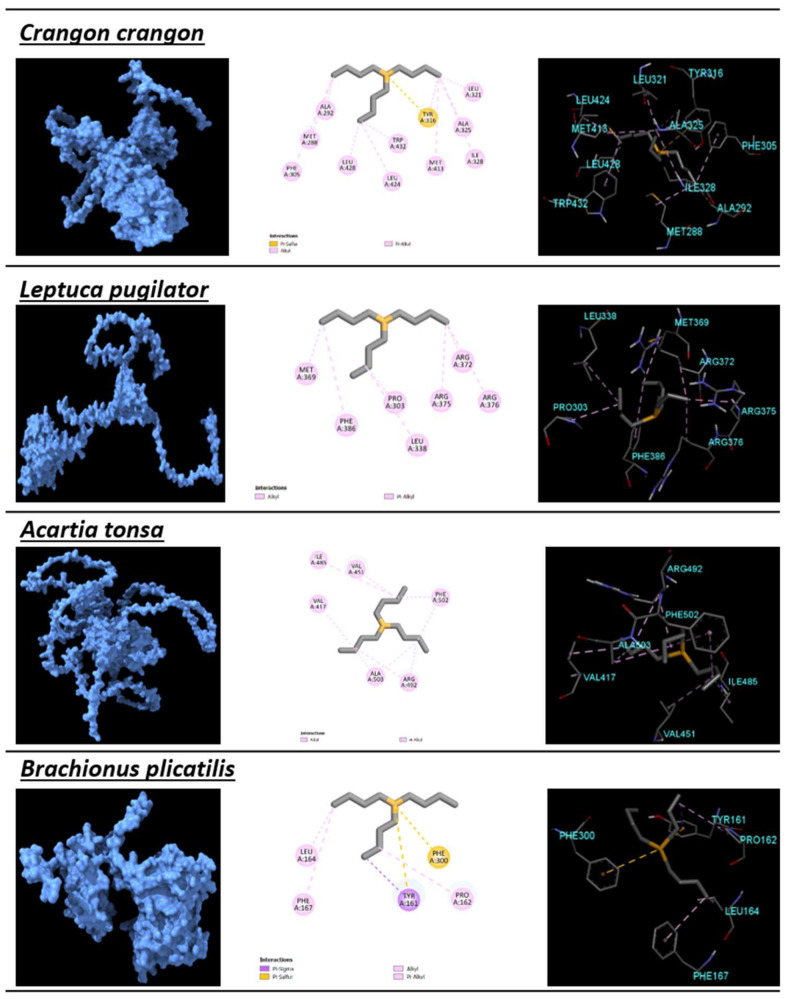
Protein models of the different species along with 2D interaction amino acids obtained from the best protein–ligand docking fit.

In models, the lower quality estimations were observed mainly for loops and terminal tails, with the docking area of the protein being well preserved. The species protein model and 2D interaction amino acids are shown in Figure 4. The docking scores (Root-Mean-Square Deviation—RMSD, inhibition constant—KI, and ligand efficiency), interaction and pocket amino acids, and rankings within the docking analysis and SSD curve are shown in Table 3. Please note that the lower the RMSD, the lower the expected inhibition constant and higher ligand efficiency. Docking was ranked from 1 to 16. The value of 1 represents the docking with the lower RMSD and inhibition constant and higher affinity, and is therefore the most sensitive species (in this case, *L. stagnalis*). As for the SSD, the species were also ranked from 1 (the most sensitive species—*C. pipiens*) to 16 (the most resistant species).

## 4. Discussion

The present study intended to analyse the possible relationship between TBT toxicity and its affinity to EcR. An SSD curve was built based on data for adult aquatic species with exposures of less than seven days. This curve enabled calculating an HC_05_ value and the different species’ positions according to their sensitivity to TBT. Although following any pattern within the curve is difficult, most vertebrate species were found within its exponential area. In contrast, invertebrates were considered the most sensitive or resistant species to TBT-based compounds. Although there is an overall perception that molluscs are the most sensitive group, the results showed that they were in the middle/upper area of the curve (most resistant species). One of the possible explanations for these results may be the different homologs proteins that TBT targets (RXR for vertebrates and molluscs, USP for insects, and EcR for crustaceans and other invertebrates) [13]. These homologs proteins play different roles within the organisms. Therefore, different pathways and functionalities within the different taxa are expected. By assessing the LC_50_ value for *T. longicaudatus*, this species can be considered one of the most resistant arthropods in the literature. Additionally, *Leptuca pugilator* appears to be the most resistant (LC_50_ = 0.8 mg/L) [27], while the arthropod *C. pipiens* showed a value (LC_50_ = 0.0029 mg/L) [28] very close to the HC_05_. Other model organisms, such as *Daphnia magna*, showed intermediary values (LC_50_ = 0.0059 mg/L) [29]. It was also expected that species with higher body masses would require higher concentrations of any given stressor to cause the same mortality rate [30]. In addition, it is crucial to notice the nature of the SSD curve, which in some cases was built with species showing a broad spectrum of values from different studies (e.g., *Tubifex tubifex* ranged from 0.1 to 10 µg/L) [31,32]. For other species, only information from one study was available (e.g., this study with the LC_50_ value for *T. longicaudatus*). Here, it is important to highlight that the adsorptive nature of TBT may result in easy contamination of the equipment used on non flow-through exposures, thus altering the actual exposure concentrations [4]. To our knowledge, the SSD curve in this study is the first to be constructed with LC_50_ values. The previous study by Lagardic et al. [4] already presented an NOEC-based SSD curve with HC_05_ = 0.39 [0.08–1.19] ng/L and a LOEC-based SSD curve with HC_05_ = 1.04 [0.3–2.62] ng/L. As expected, both curves showed an order of magnitude lower than the one observed in this study, HC_05_ = 264.9 [78.9–708.2] ng/L, thus placing it also one order of magnitude higher than the EU and USEPA freshwater thresholds of 1.5 ng/L (Directive 2013/39/EU, 12 August 2013) and 72 ng/L (EPA 822-R-03-031, 12/2003), respectively. The difference in magnitude between legislative thresholds or environmental TBT concentrations and the LC_50_ values is the reason why mortality events as a result of TBT exposure are rare or even inexistent since its ban. The literature considers that the most concerning consequences of TBT exposure are sublethal responses, with imposex (the development of male sexual organs in females) being the most studied impact [33,34,35,36]. At a molecular level, the TBT concentrations required to cause molecular initiating events are extremely low compared to those that cause basal toxicity. TBT nanomolar concentrations are able to activate RXR [37]. The interactive nature of TBT makes this molecule a non-classical endocrine disruptor, as the main impacted pathways are related to RXR and PPAR, but also to other steroid receptors (e.g., EcR, USP) that may be specific for different taxa or that may form different types of heterodimers [4]. TBT is also known to affect cytochrome P450s (CYPs) or phase II metabolism [4,38,39] and may even cause neurotoxicity by secretion of the neuropeptide APGW-amide, which is responsible for regulating male sexual differentiation in molluscs [40]. Jordão et al. [41] showed that TBT induced obesogenic effects in *D. magna* and lowered the fitness of offspring and adults by disrupting the dynamics of neutral lipids. Simões et al. [42] observed the impact of TBT on lipids but also on the oxygen carrier proteins in the haemolymph and heart glycogen. These mentioned impacts are only some of those reported in the literature. However, many other impacts, such as growth, metamorphosis, reproduction, and sexual morphism, can potentially explain the observed results [36]. Depending on the evaluated endpoints, TBT seems to display U- or inverted U-shaped responses [4,14,43].

As previously described, RXR-α and its homologs (main targets of TBT) have been found in various species across the animal kingdom [4,12]. When looking specifically at crustaceans (including the species *T. longicaudatus*), TBT’s main mode of action may be interactions with the heterodimer EcR-RXR [44,45]. The observed impact in ecdysteroids leads to changes in vitellogenesis regulation, thus retarding moult, limb regeneration abnormalities, ecdysis, and calcium reuptake disruption, leading to an overall inhibition of the exoskeleton [46,47,48]. As expected, the protein’s most conserved section includes the domains related to DNA binding and ligand binding [49]. The sequence responsible for encoding these domains is in the central section or interface of the protein sequence, which is also less prone to variation when compared to the peripherical region [50]. The interaction amino acids between TBT and human RXR-α were previously reported by Le Maire et al. (2009) [25]. For species whose protein sequences could be found within the databases, an alignment with human RXR-α was performed. The results showed two distinct groups within the molluscs. One group was very dissimilar with human RXR-α (*Biomphalaria glabrata*, *Physella acuta,* and *Ostrea edulis*) and another group where all of the interaction amino acids were similar (*L. stagnalis*, *M. edulis,* and *C. gigas*). Almost all arthropods were very dissimilar to human RXR-α, with *T. longicaudatus* showing the most similar sequence with a low replacement score (evident in the tree—Figure 3). Still, no grouping similarity could be found within the SSD curve. CYS432, which is described as crucial for TBT’s affinity to the protein, was only conserved for *L. stagnalis*, *M. edulis*, *C. gigas,* and *T. longicaudatus*, species that appear close together on the upper part of the SSD curve (Figure 1). 

Since the binding pocket is conserved across species, it was possible to perform a docking protein–ligand analysis [51]. Protein–ligand docking could be performed due to the sequences’ excellent superimposition into the used templates. The docking rank (RMSD, KI, and ligand efficiency) did not show a similar pattern to the SSD rank, showing even an opposite trend. This was unexpected, and the reasons presented previously for the results obtained in the SSD curve (e.g., LC_50_ with high ranges or lower body weight showing more resistance) may help to explain this opposite trend. Another important observation was that the key binding between CYS432 and TBT’s Sn [25] did not occur in all of the best-proposed dockings. Strong Pi–sulfur interactions between TBT’s Sn and the protein occurred for *P. acuta* (PHE277), *M. edulis* (PHE341), *D. magna* (TYR602), *Crangon crangon* (TYR316), and *Brachionus picatilis* (TYR161 and PHE300). In addition, *B. glabrata* (TYR248) also showed a pi–orbital interaction with TBT’s Sn. In either case, the strong bond did not contribute to higher affinity. Although such results require laboratory confirmation, it appears clear that other amino acids that tend to form sulfur bonds are important but do not show any relationship in regard to lower RMSD values (best affinities), the number of interaction or pocket amino acids, or even the sensitivity of the organism to TBT. 

From here, it is possible to state that although EcR is one of the main TBT targets, endocrine disruption alone is probably not responsible for the high mortality consequent to contamination-associated exposure, as identified previously by other authors. Otherwise, organisms with a high level of affinity would be on the sensitive part of the spectrum, a response that was not observable in the SSD curve. At this point, the results suggest that endocrine disruption may operate mainly as a sub-lethal effect, so mortality might be achieved by impairing one or several other pathways, leading to a cascade of events that ultimately result in the organism’s death.

## 5. Conclusions

This study approached the use of protein–ligand modeling as a possible tool to assess the sensitivity of organisms towards a stressor. The species *T. longicaudatus* has great potential to become an ecotoxicological model and thus was the crustacean with more emphasis in the present study. The aim of the study was reached by initially calculating an SSD curve with the LC_50_ reported for several invertebrate and vertebrate species (including the LC_50_ for *T. longicaudatus* determined in the present study). This was followed by protein sequence alignment and protein–ligand docking of the EcR protein from several species belonging to the SSD curve. The sequence comparison allowed us to find different clusters that could dictate the sensitivity to TBT molecules. Still, these clusters would not fit the data provided by the SSD curve. Finally, protein–ligand modeling was performed to obtain a ranking of the affinity between TBT and EcR. This analysis made it challenging to find a clear pattern, with an opposite trend being observed in the SSD curve. These findings suggest that, at the moment and without laboratory confirmation, this technique may require more than just these initial steps. The reasons for these differences may be based on the data used to build the SSD curve, the nature and characteristics of TBT, and the pathways impacted by this endocrine disruptor. Future work still needs to be developed to confirm LC_50_ values in the laboratory regarding the protein structure and TBT affinity and develop new modeling methodologies that can prove/disprove the methodology used in this study. The successful accomplishment of this work will impact the 3Rs initiative by reducing the number of organisms used in toxicological tests. 

## Figures and Tables

**Table 1:** Species with corresponding accession numbers from the different databases from which they were retrieved, along with the SWISS-MODEL template used for building the protein.

Taxa	Species	Database	Accession Number	SWISS-MODEL
Chordata	*Homo sapiens*	UniProt	P19793	Q05343.1.A
Arthropoda	*Acartia tonsa*	UniProt	A0A4Y5X195	A0A4Y5X195.1.A
	*Americamysis bahia*	NCBI	DD410574	C3UZC9.1.A
	*Crangon crangon*	UniProt	C4N541	A0A171LJQ0.1.A
	*Culex pipiens*	UniProt	A0A8D8KFP5	A4F2F2.1.A
	*Daphnia magna*	UniProt	A4F2F2	A4F2F2.1.A
	*Hyalella azteca*	UniProt	A0A979FUF3	A0A6A0GRD0.1.A
	*Leptuca pugilator*	UniProt	O76246	U6BGA5.1.A
	*Triops longicaudatus*	THIS STUDY		E9HV90.1.A
Cnidaria	*Hydra vulgaris*	UniProt	A0A8B7EC76	T2MGI4.1.A
Mollusca	*Biomphalaria glabrata*	UniProt	A0A182ZWX8	A0A182ZWX8.1.A
	*Crassostrea gigas*	UniProt	K1PXX3	K1PXX3.1.A
	*Lymnaea stagnalis*	UniProt	Q5I7G2	Q8T5C6.1.A
	*Mytilus edulis*	UniProt	A0A8S3SHA1	A0A6J8CUS3.1.A
	*Ostrea edulis*	NCBI	XM_048899976	A0A6M2XXE3.1.A
	*Physella acuta*	NCBI	GHAL01006477	A0A433TMS9.1.A
Rotifera	*Brachionus plicatilis*	UniProt	A0A3M7RAV1	A0A221CB62.1.A

**Table 2:** Interaction residues between the human protein and TBT-based compounds. The cystein responsible for the covalent bond to the tin atom of the TBT molecule is in bold.

Species		VAL	ILE	TRP	ASN	LEU	PHE	ILE	VAL	ILE	PHE	VAL	**CYS**	HIS	LEU	PHE
		265	268	305	306	309	313	324	342	345	346	349	**432**	435	436	439
*Homo sapiens*	Reference	V	I	W	N	L	F	I	V	I	F	V	C	H	L	F
*Hydra vulgaris*	Cnidaria	F	I	W	K	F	V	H	F	V	R	I	V	Q	L	V
*Biomphalaria glabrata*	Mollusca	I	M	T	T	M	A	I	L	Y	A	L	H	V	L	L
*Physella acuta*	Mollusca	V	M	S	T	M	A	I	F	Y	A	L	H	V	L	W
*Ostrea edulis*	Mollusca	L	L	S	S	M	A	I	Q	Y	T	L	H	M	L	L
*Lymnaea stagnalis*	Mollusca	V	I	W	N	L	F	I	V	I	F	V	C	H	L	F
*Mytilus edulis*	Mollusca	V	I	W	N	L	F	I	V	I	F	V	C	H	L	F
*Crassostrea gigas*	Mollusca	V	I	W	N	L	F	I	V	I	F	V	C	H	L	F
*Triops longicaudatus*	Arthropoda	L	I	W	N	L	F	I	V	I	F	V	C	H	F	F
*Hyalella azteca*	Arthropoda	F	I	T	Q	I	A	I	M	I	T	V	N	Q	C	V
*Daphnia magna*	Arthropoda	F	I	S	S	M	C	I	V	T	A	S	N	M	C	L
*Americamysis bahia*	Arthropoda	F	I	S	S	M	A	I	M	A	T	E	N	Q	C	I
*Culex pipiens*	Arthropoda	F	I	S	S	M	M	I	M	T	I	D	N	M	C	L
*Crangon crangon*	Arthropoda	F	I	S	S	M	A	I	L	S	A	A	N	M	C	L
*Leptuca pugilator*	Arthropoda	F	I	S	S	M	A	I	L	S	A	I	N	M	C	L
*Acartia tonsa*	Arthropoda	I	M	S	S	I	T	I	I	D	M	E	N	F	S	L
*Brachionus plicatilis*	Rotifera	W	F	I	F	Y	V	I	I	L	T	R	I	K	C	L

**Table 3:** Species docking scores: root mean square deviation (RMSD), inhibition constant (KI), and ligand efficiency, along with the interaction and pocket amino acids obtained for the different species. Ranking within the docking analysis and species sensitivity distribution (SSD) curve is also provided, where a lower ranking value means the species is more sensitive according to the SSD curve or docking results.

Species		RMSD	Inhibition Const.(µM)	Ligand Efficiency	Interaction Amino Acids	Pocket Amino Acids	SSD Rank	Docking Rank
***Hydra vulgaris***	**Cnidaria**	**−5.11**	179.67	0.39	PHE317, LEU387, ARG425, VAL429, PRO431, PHE434	ALA382, GLN383, CYS384, PRO385, LEU424, HIS428	3	14
***Biomphalaria glabrata***	**Mollusca**	**−5.85**	51.71	0.45	PHE138, TYR139, TYR142, LEU146, TRP205, TYR248, CYS251, LEU252	SER143, LEU202, GLU201	10	6
***Physella acuta***	**Mollusca**	**−6.07**	35.53	0.47	PHE207, ILE210, MET244, ARG247, ALA248, PHE277, TYR280, LEU284, ILE361, HIS365, ILE385, LEU389	THR241, SER245, PHE261	16	4
***Ostrea edulis***	**Mollusca**	**−5.39**	111.63	0.41	PHE249, ILE283, ARG320, ALA321, ARG323, MET324, ALA335	GLU248, PRO250, MET317, ILE332, LEU334, MET379	13	11
***Lymnaea stagnalis***	**Mollusca**	**−7.12**	6.06	0.55	VAL224, PRO226, ALA246, LEU253, TRP256, LEU283, PHE287	GLU221,ASP225, ALA245, GLN249, THR252, GLY286, ARG290, ALA301, ARG345	9	1
***Mytilus edulis***	**Mollusca**	**−5.81**	55.09	0.45	ILE296, CYS297, ALA299, ALA300, TRP333, PHE341, LEU354, ALA355, CYS460, LEU461, LEU464	GLN303, ASN334, LEU337, ILE338, ARG344, LEU479	8	8
***Crassostrea gigas***	**Mollusca**	**−6.77**	10.91	0.52	VAL233, PRO235, ALA255, LEU262, LEU292, PHE296	GLU230, GLU234, ALA254, GLN258, THR261, TRP265, GLY295, ARG299, ARG354	14	2
***Triops longicaudatus***	**Arthropoda**	**−6.31**	23.75	0.49	ALA204, TRP238, LEU242, ILE243, PHE246, ALA260, VAL282, CYS365, PHE369	ILE201, THR205, GLN208, ASN239, LEU259	12	3
***Hyalella azteca***	**Arthropoda**	**−584**	52.7	0.45	VAL455, PHE456, ILE529, ALA533, ARG536, ILE544, LEU570	ILE488, MET491, THR492, GLU454, THR495, MET530, ARG532, SER547, TYR557	6	7
***Daphnia magna***	**Arthropoda**	**−5.07**	191.61	0.39	MET536, CYS578, PHE591, TYR602, Val 607, LEU614, MET698, LEU702, LEU709	PHE530, ILE533,THR534, THR537, THR540, MET574, MET575, THR610, ASN695, CYS699, LEU713, TRP717	5	15
***Americamysis bahia***	**Arthropoda**	**−5.65**	71.6	0.43	LEU356, PRO357, ILE386, MET424, ALA428, ILE439, TYR441	GLU355, ALA387, ALA390, LEU393, ARG427, ARG431, LEU440, PHE442, TYR452, LEU465	4	9
***Culex pipiens***	**Arthropoda**	**−5.59**	79.39	0.43	PRO315, PRO316, ALA319, LYS349, TRP352, TYR353	CYS312, ASP313, PRO314, HIS317, ALA345, TYR348	1	10
***Crangon crangon***	**Arthropoda**	**−5.12**	176.12	0.39	MET288, ALA292, PHE305, TYR316, LEU321, ALA325, ILE328, MET413, LEU424, LEU428, TRP432	ILE247, TYR248, THR251, TYR254, MET289, SER324	11	13
***Leptuca pugilator***	**Arthropoda**	**−5.88**	48.62	0.45	MET369, MET370, ALA373, TYR397, LEU402, LEU409, MET494, TRP513	ILE328, THR329, THR332, ILE384, PHE386, SER405, ALA406, CYS495, LEU498, LEU505, LEU509	15	5
***Acartia tonsa***	**Arthropoda**	**−5.26**	140.33	0.4	VAL417, VAL451, ILE485, ARG492, PHE502, ALA503	GLU416, PRO418, MET444, SER447, THR448, ARG488, THR489, VAL501	2	12
***Brachionus plicatilis***	**Rotifera**	**−4.54**	246.87	0.38	TYR161, PRO162, LEU164, PHE167, PHE300	LYS159, ASP163, TYR166, ILE301, ASN302, GLY303, ALA337, ASN340	7	16

## Data Availability

Data are contained within the article.
